# Comparison of outpatient antibiotic prescriptions among older adults in IQVIA Xponent and publicly available Medicare Part D data, 2018

**DOI:** 10.1017/ash.2022.332

**Published:** 2023-02-15

**Authors:** Elizabeth M. Beshearse, Katryna A. Gouin, Katherine E. Fleming-Dutra, Sharon Tsay, Lauri A. Hicks, Sarah Kabbani

**Affiliations:** 1 Centers for Disease Control and Prevention, Atlanta, Georgia; 2 Epidemic Intelligence Service, Centers for Disease Control and Prevention, Atlanta, Georgia; 3 Chenega Corporation, contractor on assignment to the National Center for Emerging and Zoonotic Infectious Diseases, US Centers for Disease Control and Prevention, Atlanta, Georgia

**Keywords:** antibiotic prescribing, outpatient, older adults

## Abstract

The distributions of antibiotic prescriptions by geography, antibiotic class, and prescriber specialty are similar in the US Centers for Medicare and Medicaid Services (CMS) Part D Prescriber Public Use Files and IQVIA Xponent dataset. Public health organizations and healthcare systems can use these data to track antibiotic use and guide antibiotic stewardship interventions for older adults.

In 2014–2016, the highest rates of outpatient antibiotic prescribing were observed in adults 65 years and older; 30% of antibiotic prescriptions in this population are considered unnecessary.^1,2^ The Centers for Disease Control and Prevention (CDC) Core Elements of Outpatient Antibiotic Stewardship highlight the importance of tracking and reporting of clinician prescribing.^3^ Proprietary databases, such as IQVIA Xponent,^1,4–7^ have been used for tracking antibiotic use at the national level and identifying opportunities for improving prescribing practices, but require funding and have not been compared with other data sources that characterize antibiotic prescribing. Stakeholders can use prescription data from the Centers for Medicare and Medicaid Services (CMS) at low to no cost to support antibiotic stewardship activities.^8^ The objectives of this analysis were to evaluate the three publicly available CMS Part D Prescriber Public Use Files (PUFs) and compare to the IQVIA Xponent dataset to serve as a guide and resource for public health organizations and healthcare systems considering using these data to improve antibiotic use.

## Methods

**Fig. 1.**
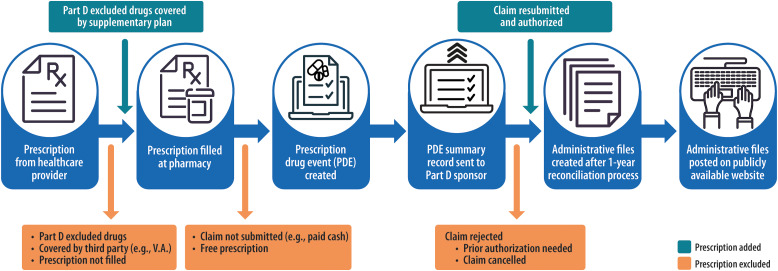
Centers for Medicare and Medicaid Services (CMS) Part D prescription drug event (PDE) reconciliation process. Note. Other CMS administrative datasets that are available to research include the Virtual Research Data Center (VRDC) Chronic Conditions Warehouse (CCW) and limited datasets.

Adults 65 years and older, as well as individuals under 65 with certain medical conditions, are eligible to enroll in Medicare insurance coverage. Approximately 94% of adults 65 and older are enrolled in Medicare and 72% have Part D prescription coverage.^9^ The CMS Part D Prescriber PUFs capture 100% of Part D final-action prescription drug event (PDE) records (Fig. 1). These data identify prescribers by their national provider identifier (NPI) and provide a variable to indicate antibiotics as defined by CMS.^8^ PDE data are released annually when claims are finalized with a 2-year lag.^8^

The CMS provide three Part D Prescriber PUFs that are publicly available and contain information on drug utilization: Geography-Drug, Provider, and Provider-Drug datasets.^8^ The Part D Prescriber PUFs offer data characteristics that vary depending on the level of aggregation. The Geography-Drug dataset contains counts of prescription drug claims aggregated at the national and state levels. Prescriber-level information, including specialty, cannot be assessed using the Geography-Drug dataset. The Provider dataset contains a count of total prescription drug claims aggregated at the prescriber level and includes prescriber characteristics (name, NPI, specialty, ZIP code) but antibiotic agent and class cannot be assessed. The Provider-Drug dataset contains counts of total prescription drug claims and is organized by individual prescriber and specific drug. A drug with <11 prescriptions is suppressed; as a result, there is higher suppression at the prescriber level and total antibiotic claim counts are not equal across all four datasets.

IQVIA Xponent has been used to describe national outpatient antibiotic prescribing.^1,4–7^ In 2018, IQVIA Xponent captured ∼92% of the outpatient prescriptions that were dispensed by retail pharmacies in the United States.^1^ IQVIA then uses a proprietary projection methodology to estimate 100% of outpatient prescriptions.^5^

We determined the antibiotic data elements available and assessed the level of suppression of prescription claims by comparing the overall volume of antibiotic prescriptions in each dataset to the national level of the Geography-Drug dataset in 2018. We compared the distribution of antibiotic claim counts by US Census region, antibiotic class, and prescriber specialty when available. The Geography-Drug, Provider, and Provider-Drug datasets were stratified by state. The Geography-Drug and Provider-Drug datasets were stratified by antibiotic class and agent.

We limited the comparison of CMS Part D Prescriber PUF and IQVIA Xponent to adults aged ≥65, included only oral antibiotics available in both datasets and excluded non–US states (eg, territories or armed forces locations). We used the Geography-Drug dataset at the state level for comparison since data could be stratified by age, region, and antibiotic class with minimal suppression. This activity met the requirements of public health surveillance as defined in 45 CFR 46.102(l)(2).

## Results

Across the CMS Part D Prescriber PUF datasets, the number of outpatient antibiotic prescriptions ranged from 64.6 million in the national level of the Geography-Drug dataset to 44.5 million in the Provider-Drug dataset (Table 1). The Geography-Drug dataset aggregated at the geographic level has minimal suppression: only drugs <11 prescriptions are suppressed. In contrast, the Provider and Provider-Drug datasets aggregated at the prescriber level have 10% and 31% fewer antibiotic prescriptions than the national level of the Geography-Drug dataset, respectively. We also detected a 28% decrease in number of providers from the Provider dataset (N = 679,880) to the Provider-Drug dataset (N = 488,660) due to data suppression. Nonetheless, the distribution of antibiotic prescriptions across the datasets varied by <2% with respect to region, antibiotic class, and prescriber specialty (Table 1).

**Table 1.** Comparison of Number of Antibiotic Prescriptions by US Census Region, Antibiotic Class, and Specialty Across Centers for Medicare and Medicaid Services (CMS) Part D Prescriber Public Use Files^a^ and Number of Oral Antibiotic Prescriptions Among Adults Aged ≥65 Years by US Census Region, Prescriber Specialty, Antibiotic Class, and Antibiotic Agent Between CMS Part D Prescriber Public Use Files and IQVIA Xponent, 2018

	CMS Part D Prescriber Public Use Files										IQVIA Xponent	
	Antibiotic Prescriptions for Medicare Part D Beneficiaries								Oral Antibiotic Prescriptions for Those Aged ≥65 Years			
Data Source	Geography-Drug National Level		Geography-Drug State^b^ Level		Provider		Provider-Drug		Geography-Drug State^b^ Level		IQVIA Xponent	
Characteristic	No., millions	%	No., millions	%	No., millions	%	No., millions	%	No., millions	%	No., millions	%
Total	64.6		63.7		57.9		44.5		48.4		57.6	
**US Census region**	Cannot be assessed											
South			26.3	(41)	24.3	(42)	19.3	(43)	19.7	(41)	24.4	(42)
Midwest			14.2	(22)	13.0	(23)	9.8	(22)	10.8	(22)	12.6	(22)
Northeast			11.6	(18)	10.3	(18)	7.8	(17)	8.9	(18)	10.6	(18)
West			11.6	(18)	10.3	(18)	7.6	(17)	9.0	(19)	10.0	(17)
**Antibiotic class**					Cannot be assessed							
Fluoroquinolones	10.9	(17)	10.6	(17)			7.7	(17)	8.0	(17)	9.1	(16)
Cephalosporins	9.7	(15)	9.6	(15)			6.8	(15)	7.8	(16)	9.1	(16)
Macrolides	9.4	(15)	9.2	(15)			7.3	(16)	7.6	(16)	8.5	(15)
Penicillins	8.3	(13)	8.3	(13)			6.5	(14)	6.9	(14)	9.4	(16)
Tetracyclines	5.9	(9)	5.9	(9)			4.0	(9)	4.8	(10)	5.8	(10)
Trimethoprim-sulfamethoxazole	5.2	(8)	5.1	(8)			3.6	(8)	4.0	(8)	4.7	(8)
B-lactams, increased activity	5.3	(8)	5.2	(8)			3.5	(8)	3.9	(8)	4.9	(9)
Urinary anti-infectives	3.4	(5)	3.4	(5)			2.1	(5)	2.9	(6)	3.4	(6)
Other^c,d^	3.6	(6)	3.5	(6)			1.6	(4)	0.4	(1)	0.2	(0.4)
Lincosamides	2.9	(4)	2.8	(4)			1.4	(3)	2.1	(4)	2.4	(4)
**Specialty** ^e^	Cannot be assessed		Cannot be assessed						Cannot be assessed			
Family practice					12.3	(21)	9.5	(21)			11.7	(20)
Internal medicine					11.5	(20)	8.8	(20)			9.5	(17)
Nurse practitioner					7.8	(13)	5.1	(12)			7.3	(13)
Dentist					5.3	(9)	4.4	(10)			7.0	(12)
Physician assistant					4.8	(8)	3.2	(7)			5.0	(9)
Emergency medicine					2.7	(5)	1.4	(3)			2.2	(4)
Urology					2.5	(4)	2.3	(5)			2.8	(5)

Note. CMS, Centers for Medicare and Medicaid Services.

a The CMS Part D Prescriber Public Use Files can be accessed and downloaded at https://data.cms.gov/provider-summary-by-type-of-service/medicare-part-d-prescribers.

b We excluded the following locations from the analysis of the CMS Part D Prescriber Geography-Drug dataset: Armed Forces Central/South America, Armed Forces Europe, Armed Forces Pacific, American Samoa, Foreign Country, Guam, Northern Mariana Islands, Puerto Rico, Virgin Islands, Unknown.

c The “other” antibiotic class in the comparison across CMS Part D Prescriber Public Use Files includes antibiotic agents: amikacin, dalbavancin, daptomycin, gentamicin, linezolid, metronidazole, neomycin sulfate, oritavancin, quinupristin/dalfopristine, rifaximin, secnidazole, streptomycin, tedizolid, telavancin, tigecycline, tinidazole, tobramycin, vancomycin.

d The “other” antibiotic class in the CMS and IQVIA Xponent comparison includes oral antibiotics present in both data sources: linezolid, tedizolid, vancomycin.

e Top 7 prescriber specialties by antibiotic claim count represented.

For adults aged ≥65 years, the number of oral antibiotic prescriptions was lower in the CMS Part D Geography-Drug dataset at the state level (N = 48.4 million) compared to the IQVIA Xponent dataset (N = 57.6 million). The proportion of antibiotic prescriptions by region and antibiotic class varied by <2% across both datasets (Table 1). Stratification by specialty was not available in the Geography-Drug dataset so it could not be compared to IQVIA Xponent.

## Discussion

The distribution of prescriptions by geography, antibiotic class, and prescriber specialty in the CMS Part D Prescriber PUFs are similar to the distributions in the national IQVIA Xponent dataset. Furthermore, the CMS Part D Prescriber PUFs have been used previously to describe national and regional outpatient antibiotic prescribing trends.^10^ CMS Part D Prescriber PUFs are publicly available and readily accessible to public health organizations and healthcare systems.

The CMS Part D Prescriber PUFs include unique data characteristics that must be considered to determine which dataset best aligns with program goals and stewardship interventions.^3^ The Geography-Drug dataset can be used to describe annual prescription trends by antibiotic class and agent among older adults enrolled in Medicare Part D but cannot be used to provide prescriber feedback. The Provider dataset can be used to describe antibiotic prescription trends by geographic region (including state and city) and provider specialty. Although antibiotic class and agent cannot be described in the Provider dataset, it can be used to assess prescriber-level total antibiotic volume with the least analytic manipulation. The Provider-Drug dataset provides the most detail at the prescription level. However, it has the highest level of data suppression and its complexity requires more analysis to describe prescriber-level prescribing practices compared to the Provider dataset. Individualized feedback provided to clinicians on antibiotic prescribing practices, especially when including comparison with peers, has been shown to be effective at reducing antibiotic prescribing in high-volume antibiotic prescribers.^7^ The Provider dataset may be most suitable for public health organizations and healthcare systems to provide prescriber feedback.^9^

There were differences in antibiotic volume between the CMS Part D Prescriber PUFs and IQVIA Xponent. IQVIA Xponent is projected to 100% of the retail market, whereas Part D prescription claims cover ∼75% of Medicare beneficiaries. Also, prescription claim counts <11 are suppressed in CMS Part D Prescriber PUFs, which has a larger impact on total antibiotic volume at the prescriber level; up to one-third of prescriptions and prescribers are suppressed in different CMS Part D Prescriber PUFs based on the level of aggregation.^9^ One limitation of both IQVIA Xponent and the CMS Part D Prescriber PUFs is that they do not contain clinical diagnoses; therefore, appropriateness of antibiotic prescribing cannot be assessed. However, these data could be a starting point to determine which providers should be targeted to assess appropriateness. Another limitation of the CMS Part D Prescriber PUFs is that the 2-year data lag may not reflect current prescribing practices.^10^

The CMS Part D Prescriber by Provider dataset provides readily available data for public health organizations and health systems to assess antibiotic prescribing among adults 65 years and older and to identify prescribers for peer comparison audit and feedback interventions.

## References

[1] King LM , Bartoces M , Fleming-Dutra KE , Roberts RM , Hicks LA. Changes in US outpatient antibiotic prescriptions from 2011–2016. Clin Infect Dis 2020;70:370–377. 30882145 10.1093/cid/ciz225 PMC8078491

[2] Hersh AL , King LM , Shapiro DJ , Hicks LA , Fleming-Dutra KE. Unnecessary antibiotic prescribing in US ambulatory care settings, 2010–2015. Clin Infect Dis 2021;72:133–137. 32484505 10.1093/cid/ciaa667 PMC9377284

[3] Sanchez GV , Fleming-Dutra KE , Roberts RM , Hicks LA. Core elements of outpatient antibiotic stewardship. Centers for Disease Control and Prevention website. https://www.cdc.gov/mmwr/volumes/65/rr/rr6506a1.htm. Published 2016. Accessed July 19, 2021.

[4] Staub MB , Ouedraogo Y , Evans CD , et al. Analysis of a high-prescribing state’s 2016 outpatient antibiotic prescriptions: implications for outpatient antimicrobial stewardship interventions. Infect Control Hosp Epidemiol 2020;41:135–142. 31755401 10.1017/ice.2019.315 PMC7309961

[5] Hicks LA , Bartoces MG , Roberts RM , et al. US outpatient antibiotic prescribing variation according to geography, patient population, and provider specialty in 2011. Clin Infect Dis 2015;60:1308–1316. 25747410 10.1093/cid/civ076

[6] Kabbani S , Palms D , Bartoces M , Stone N , Hicks LA. Outpatient antibiotic prescribing for older adults in the United States: 2011 to 2014. J Am Geriatr Soc 2018;66:1998–2002. 30221746 10.1111/jgs.15518 PMC7909599

[7] Schwartz KL , Ivers N , Langford BJ , et al. Effect of antibiotic-prescribing feedback to high-volume primary care physicians on number of antibiotic prescriptions: a randomized clinical trial. JAMA Intern Med 2021;181:1165–1173. 34228086 10.1001/jamainternmed.2021.2790 PMC8261687

[8] Centers for Medicare and Medicaid Services. Medicare Part D Prescribers. https://data.cms.gov/provider-summary-by-type-of-service/medicare-part-d-prescribers. Accessed July 13, 2021

[9] Berchick ER , Barnett JC , Upton RD. Health insurance coverage in the United States, 2018. Pp. 60–267. US Census website. https://www.census.gov/content/dam/Census/library/publications/2019/demo/p60-267.pdf. Published 2019. Accessed May 5, 2021.

[10] Arizpe A , Reveles KR , Aitken SL. Regional variation in antibiotic prescribing among medicare part D enrollees, 2013. BMC Infect Dis 2016;16:744. 27938336 10.1186/s12879-016-2091-0 PMC5148872

